# A retrospective analysis of glycol and toxic alcohol ingestion: utility of anion and osmolal gaps

**DOI:** 10.1186/1472-6890-12-1

**Published:** 2012-01-12

**Authors:** Matthew D Krasowski, Rebecca M Wilcoxon, Joel Miron

**Affiliations:** 1 Department of Pathology, University of Iowa Hospitals and Clinics, Iowa City, IA, 52242, USA

**Keywords:** Ethylene glycol, isopropanol, methanol, propylene glycol, retrospective studies, sensitivity and specificity

## Abstract

**Background:**

Patients ingesting ethylene glycol, isopropanol, methanol, and propylene glycol ('toxic alcohols') often present with non-specific signs and symptoms. Definitive diagnosis of toxic alcohols has traditionally been by gas chromatography (GC), a technique not commonly performed on-site in hospital clinical laboratories. The objectives of this retrospective study were: 1) to assess the diagnostic accuracy of the osmolal gap in screening for toxic alcohol ingestion and 2) to determine the common reasons other than toxic alcohol ingestion for elevated osmolal gaps.

**Methods:**

Electronic medical records from an academic tertiary care medical center were searched to identify all patients in the time period from January 1, 1996 to September 1, 2010 who had serum/plasma ethanol, glucose, sodium, blood urea nitrogen, and osmolality measured simultaneously, and also all patients who had GC analysis for toxic alcohols. Detailed chart review was performed on all patients with osmolal gap of 9 or greater.

**Results:**

In the study period, 20,669 patients had determination of serum/plasma ethanol and osmolal gap upon presentation to the hospitals. There were 341 patients with an osmolal gap greater than 14 (including correction for estimated contribution of ethanol) on initial presentation to the medical center. Seventy-seven patients tested positive by GC for one or more toxic alcohols; all had elevated anion gap or osmolal gap or both. Other than toxic alcohols, the most common causes for an elevated osmolal gap were recent heavy ethanol consumption with suspected alcoholic ketoacidosis, renal failure, shock, and recent administration of mannitol. Only 9 patients with osmolal gap greater than 50 and no patients with osmolal gap greater than 100 were found to be negative for toxic alcohols.

**Conclusions:**

Our study concurs with other investigations that show that osmolal gap can be a useful diagnostic test in conjunction with clinical history and physical examination.

## Background

Consumption of toxic alcohols other than ethanol continues to be a public health problem [1]. The most common toxic alcohols are ethylene glycol, isopropanol, and methanol. All three compounds are found in products that are easily obtained (ethylene glycol in most automobile antifreezes, isopropanol in 'rubbing alcohol', and methanol in windshield cleaner fluid and some other products). Ethylene glycol and methanol are particularly dangerous in overdose, due to their metabolites that can cause severe organ damage [1-6].

Ethylene glycol is metabolized by a series of steps to glycolic acids and oxalic acid, the latter with the potential to cause severe renal injury [2-5]. Methanol is likewise metabolized by a series of enzymatic reactions to formic acid, a toxic compound that can cause blindness from permanent injury to the optic nerve. Both ethylene glycol and methanol are capable of causing marked metabolic acidosis, mainly due to their metabolites. Following ingestion of ethylene glycol or methanol, an osmolal gap appears first and an anion gap appears later after conversion to acidic metabolites [1-5]. Isopropanol is generally less toxic than ethylene glycol or methanol, as it is primarily metabolized to acetone [7,8]. However, in addition to the organ damage caused by metabolites of ethylene glycol and methanol, all three toxic alcohols are capable of producing central nervous system (CNS) depression that in and of itself may be life-threatening [1,4].

The definitive laboratory method for detecting and quantitating toxic alcohols in the serum/plasma is gas chromatography (GC) [6]. However, this technique is labor-intensive and not available at most clinical laboratories associated with hospitals and medical centers, with the exception of some larger medical center laboratories. Consequently, this analysis is generally performed at remote reference laboratories, often precluding a turnaround time of 2-4 hr as recommended by a consensus panel for optimal management of patients ingesting ethylene glycol or methanol [6].

Diagnosis of toxic alcohol ingestion therefore often relies on clinical signs and symptoms along with indirect evidence from laboratory tests such as arterial blood gas analysis (to detect acidosis), serum osmolality (to estimate osmolal gap, OG), and common chemistry tests (to calculate anion gap). Prompt diagnosis of toxic alcohol poisoning can provide major benefit to patients. If diagnosed early enough, ethylene glycol and methanol poisonings are usually treated effectively by administration of either ethanol or fomepizole, both of which inhibit the rate-limiting first step in the metabolism of ethylene glycol or methanol by alcohol dehydrogenase and thus prevent the formation of toxic metabolites [2,3,9,10]. Toxic alcohol ingestions that are not diagnosed early often require hemodialysis to clear both the parent compounds and metabolites, although end-organ damage may already have occurred. Conversely, an erroneous false diagnosis of toxic alcohol ingestion has the downside of increased expense and potential adverse effects related to antidotal therapy and/or hemodialysis.

The OG is determined by measuring serum osmolality (e.g., by freezing point depression) and then using a formula to calculate the osmolality contribution of the endogenous major contributors to serum osmolality, namely sodium, blood urea nitrogen (BUN), and glucose, which are standard chemistry tests frequently ordered in patients with potential toxic alcohol ingestions [11,12]. The OG is the measured osmolality minus the estimated osmolality. There are also formulae to account for the presence of serum ethanol (if present). There is considerable debate over the use of OG to diagnose toxic alcohol ingestions, and also a plethora of formulae proposed for estimating the contribution of sodium, BUN, glucose, and ethanol to serum osmolality [8,11-29]. An elevated OG (often defined as greater than a threshold between 10 and 15) suggests the presence of osmotically active substances other than sodium, BUN, glucose, and ethanol. The differential diagnosis for elevated OG includes a variety of conditions other than toxic alcohol ingestion such as alcoholic ketoacidosis [23,30-32], mannitol infusion [33,34], renal failure [35,36], and shock [37,38]. In some of these conditions (e.g., shock), the exact osmotically active compounds are not exactly known. Alcoholic ketoacidosis can produce a substantial osmolal gap even in the absence of detectable plasma ethanol due to the formation of glycerol, acetone, and the acetone metabolites acetol and 1,2-propanediol [31].

An additional toxic alcohol compound that can cause an elevated OG is propylene glycol [1,39]. Although chemically similar to ethylene glycol (and also used in some automobile antifreezes), propylene glycol is generally much less toxic than ethylene glycol. Propylene glycol is found in a variety of products including cosmetics, ointments, some activated charcoal preparations, and as a diluent for intravenous preparations of poorly water-soluble drugs such as diazepam, etomidate, and lorazepam. Propylene glycol toxicity has been described in overdoses of propylene glycol-containing antifreeze [40]. A number of studies have detailed propylene glycol toxicity from repeated intravenous administrations of medications containing propylene glycol as the diluent, particularly lorazepam used for extended sedation (e.g., for patients who are intubated for mechanical ventilation) [41-45].

In this study, we performed a retrospective analysis of toxic alcohol and OG analyses in a timespan of nearly 15 years at a tertiary care academic medical center. The primary objectives were to assess the diagnostic accuracy of OG as a test for screening for toxic alcohol ingestion and to define the common causes of elevated OG in the absence of toxic alcohol ingestion. The study conforms to the Standards for Reporting Diagnostic Accuracy (STARD) statement criteria [46,47].

## Methods

### Setting

We conducted a retrospective analysis of electronic laboratory and medical records from a tertiary care academic medical center. An important panel of laboratory tests for this study was the 'Ethanol Volatile Panel' which included serum/plasma sodium, BUN, glucose, ethanol (by enzymatic assay), and plasma osmolality. From this panel, the OG was calculated.. This panel was commonly ordered for patients presenting with clinical histories, signs, and symptoms consistent with toxic ingestions (e.g., altered mental status, obtundation, suicide attempt, or possible accidental ingestion by a child). GC analysis for toxic alcohols or glycols required approval by pathology resident or clinical chemistry laboratory director (or cross-covering pathologist) and often occurred in the context of an Ethanol Volatile Panel showing elevated OG (> 14) after correcting for plasma ethanol. Initial GC analysis was typically run on the same plasma specimen that the Ethanol Volatile Panel was performed on unless there was insufficient specimen to do so.

### Selection of Study Subjects

The electronic medical record (Epic, Epic Systems Inc., Madison, WI, USA) was searched for the time period from 1/1/1996 to 9/1/2010 for all occurrences when the Ethanol Volatile Panel or GC analysis for toxic alcohols was ordered. The results of these tests along with patient age, gender, and medical center location at time of blood draw (e.g., emergency room, inpatient floor, outpatient clinic, etc.) were downloaded. In the event of multiple hospital visits by a single patient, only the first visit was included in the analysis. The project had Institutional Review Board approval from the University of Iowa.

**Figure 1:**
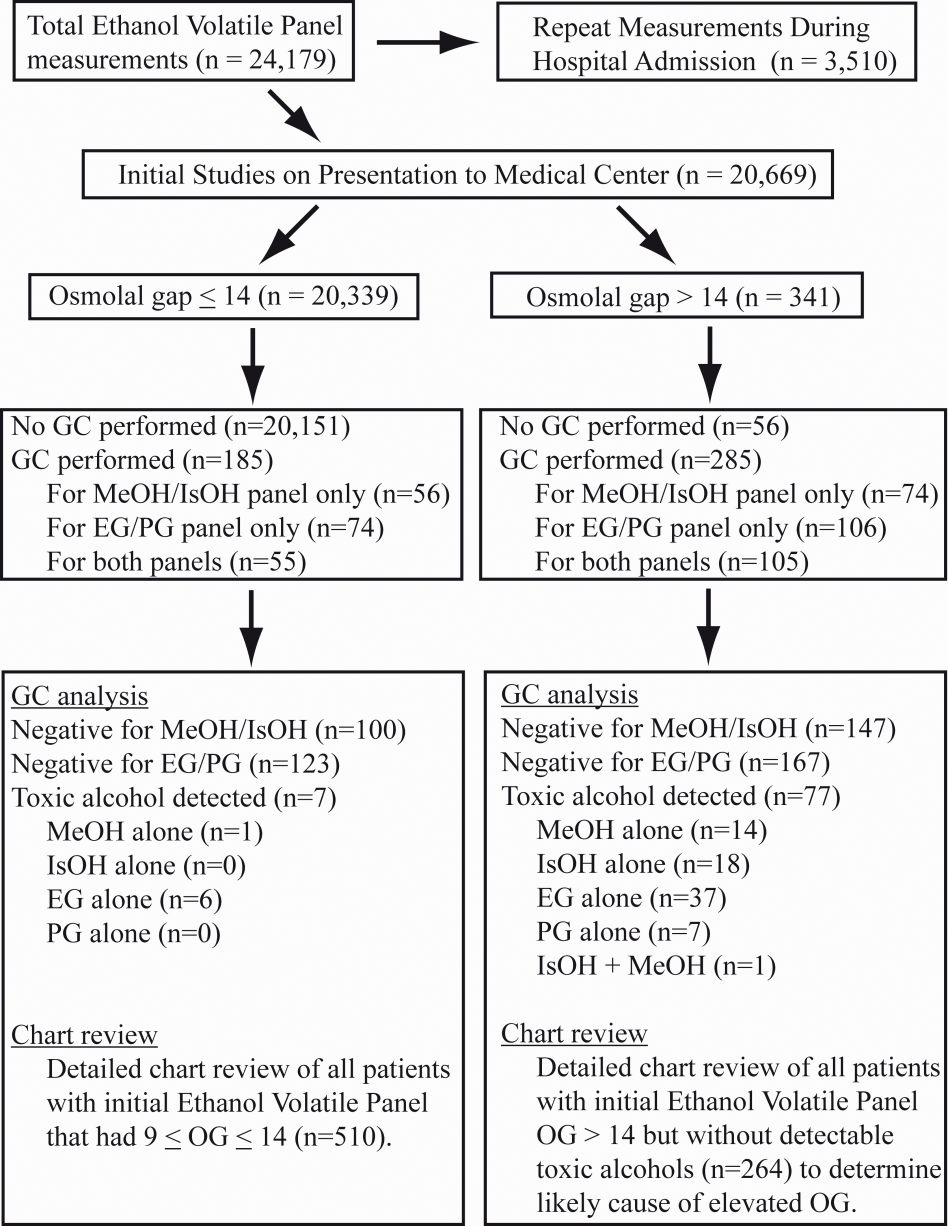
**Flow diagram illustrating the derivation of the study sample**. Abbreviations: EG, ethylene glycol; GC, gas chromatography; IsOH, isopropanol; MeOH, methanol; PG, propylene glycol.

Figure 1 shows the patient populations and subsets that were subjected to more detailed analysis. Chart review was performed on all patients with OG ≥ 9. In the case of patients with OG > 14 on initial laboratory studies but without any detection of toxic alcohols by GC, the chart review was intended to identify the likely cause of the elevated OG. For patients with OG ≥ 9 but ≤14, the chart review aimed to determine whether any patients had clinical history compatible with toxic alcohol ingestion whether or not GC was performed.

### Methods of Measurement

All laboratory measurements were performed in the central Clinical Chemistry laboratory. Serum/plasma electrolytes, BUN, glucose, β-hydroxybutyrate, and ethanol were determined on high volume chemistry analyzers (Roche P modules, Roche Diagnostics, Inc., Indianapolis, IN, USA). In addition to a specific quantitative assay for serum/plasma β-hydroxybutyrate (Stanbio Laboratory, Boerne, TX, USA), a semi-qualitative test using a colorimetric assay was also available for assessment of serum and urine ketones (Acetest^®^, Bayer Diagnostics, now part of Siemens Healthcare Diagnostics, Deerfield, IL, USA). Serum/plasma osmolality was determined by freezing point depression (Model 2020 osmometer, Advanced Instruments, Inc., Norwood, MA, USA). Serum/plasma concentrations of ethylene glycol, propylene glycol, methanol, isopropanol, and acetone were measured by GC (Agilent 6850 with 7683 injector, Agilent Technologies, Santa Clara, CA, USA), which served as the gold standard (reference) technique for diagnosis of toxic alcohol ingestion. The lower concentration limit for clinical reporting for ethanol, ethylene glycol, isopropanol, methanol, and propylene glycol was 10 mg/dL. Laboratory analyses were performed by clinical laboratory staff as part of patient care.

OG was calculated using a formula by Khajuria and Krahn [21]:OG = (Measured osmolality) - {2 × [Sodium] + (1.15 * [Glucose]/18) + ([BUN]/2.8) + (1.2 * [ETOH]/4.6)} where [Sodium] is plasma sodium concentration (in mEq/L), [Glucose] is plasma glucose concentration (in mg/dL), [BUN] = plasma blood urea nitrogen concentration (in mg/dL), and [ETOH] is plasma ethanol concentration (in mg/dL). Anion gap was equal to the plasma sodium concentration minus the sum of the plasma bicarbonate and chloride concentrations (all measured in mEq/L).

Sensitivity was defined as: (number of true positives)/(number of true positives + number of false negatives). Specificity was defined as (number of true negatives)/(number of true negatives + number of false positives). Statistical analyses were carried out in EP Evaluator release 9 (Data Innovations, South Burlington, VT, USA). Graphs were generated in Kaleidagraph version 4.0 (Synergy Software, Reading, PA, USA).

## Results

### Characteristics of Study Subjects

**Figure 2:**
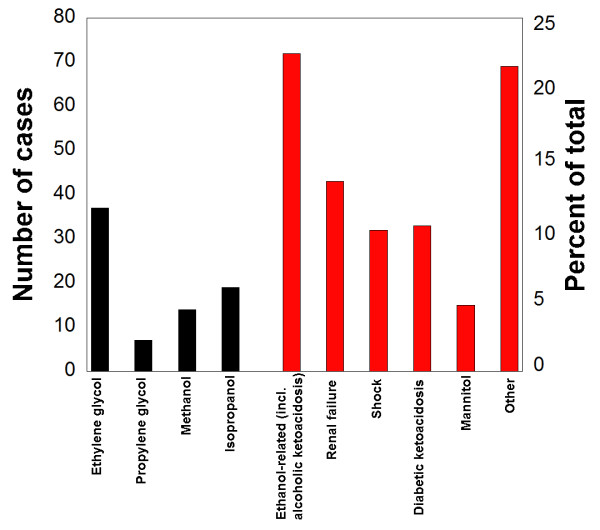
**Causes of elevated osmolal gaps**. For the 341 patients who presented with elevated osmolal gap (OG) > 14 on initial laboratory studies on presentation to the medical center, the suspected primary cause was determined from analysis of toxic alcohols by gas chromatography and by chart review. The black bars indicate number of patients where toxic alcohol ingestion was the likely primary cause of the elevated OG. The red bars are suspected primary causes of elevated OG in the absence of toxic alcohol ingestions.

We identified 341 patients that had OG greater than 14 on the Ethanol Volatile Panel determined on initial presentation (Figure 1; Table 1). Of these 341 patients, 285 had GC analysis performed, identifying 77 patients with ethylene glycol, propylene glycol, methanol, and/or isopropanol present in serum/plasma at concentrations of 10 mg/dL or greater by GC (Figure 2). GC analysis was also performed in 185 patients with OG of 14 or less (Figure 1; also discussed in more detail below). These analyses identified 6 patients with ethylene glycol and 1 patient with methanol at plasma concentrations of 10 mg/dL or greater (Table 2). There were thus a total of 84 patients with detectable toxic alcohols on initial laboratory studies.

**Table 1:** Demographics of patient populations

	N (males/females)	Average age ± SD	Age range
			

OG > 14	341 (229/112)	43.2 ± 16.3	0.9 - 89.7

No toxic alcohol detected	264 (192/72)	42.5 ± 15.8	1.8 - 89.7

Ethanol-related	72 (52/20)	46.6 ± 14.3	14.6 - 84.9

Renal failure	43 (30/13)	48.0 ± 14.0	15.4 - 89.7

Shock	32 (18/14)	43.1 ± 18.7	14.4 - 83.8

Diabetic ketoacidosis	33 (25/8)	40.9 ± 14.2	14.2 - 69.9

Mannitol	15 (10/5)	37.9 ± 22.3	1.8 - 80.4

Other	69 (47/22)	35.7 ± 13.2	15.7 - 62.8

			

Toxic alcohol detected	77 (47/30)	35.5 ± 16.5	1.4 - 72.1

Ethylene glycol	37 (25/12)	35.7 ± 14.1	19.0 - 72.1

Isopropanol	19 (8/11)	30.5 ± 21.5	1.4 - 64.6

Methanol	14 (11/3)	42.3 ± 13.7	20.2 - 71.4

Propylene glycol	7 (3/4)	41.0 ± 9.6	25.6 - 54.5


**Table 2:** Summary of toxic alcohol ingestions without elevated osmolal gap

Age	Gender	Alcohol and glycol serum concentration	Clinical history	Anion gap	Osmolal gap
2	M	Methanol 12 mg/dL	Ingestion of small amount of windshield fluid, brought quickly to emergency room	16	-5

19	F	Ethylene glycol 18 mg/dL	Intentional ingestion of ~4 ounces of antifreeze 12 hours prior to presentation	28	2

24	F	Ethylene glycol 89 mg/dL	Intentional ingestion of unknown amount of antifreeze unknown time before presentation	27	5

32	M	Ethylene glycol 44 mg/dL Ethanol 113 mg/dL	Accidental ingestion of antifreeze 45 mins prior to emergency room visit	Not determined	6

39	F	Ethylene glycol 30 mg/dL Ethanol 83 mg/dL	Intentional ingestion of 8 ounces of antifreeze 6 hours prior to presentation	15	7

40	M	Ethylene glycol 27 mg/dL Ethanol 202 mg/dL	Intentional ingestion of 4-5 "mouthfuls" of antifreeze unknown time before presentation	17	7

44	F	Ethylene glycol 32 mg/dL Ethanol 270 mg/dL	Intentional ingestion of 1 cup of antifreeze 1 hour prior to presentation	15	-1

**Figure 3:**
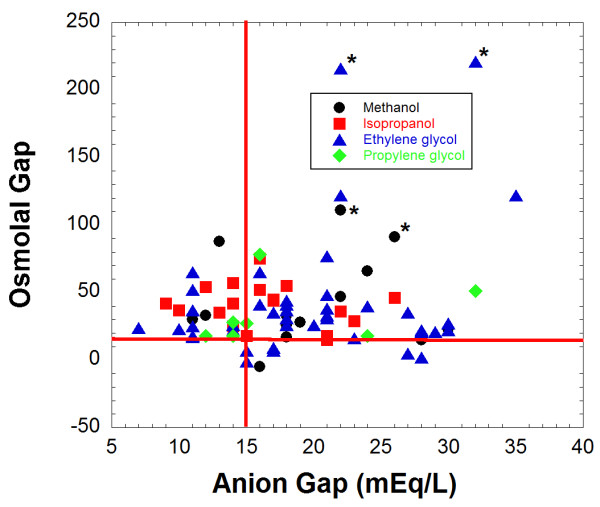
**Osmolal and anion gaps for patients who had ingested toxic alcohols**. For patients who had ethylene glycol, isopropanol, or methanol detected by gas chromatography analysis, the osmolal gap and anion gap on initial laboratory studies were plotted. This plot includes a total of 73 patients. Eleven patients with toxic alcohols detected by GC were not included due to lack of laboratory data necessary to calculate anion gap. The data is sorted by the primary ingestion. A single case of co-ingestion of isopropanol and methanol is classified with the isopropanol group as that compound was detected in higher amount in serum for that patient. Two fatal cases each for ethylene glycol and methanol (total of 4 patients) are designated by an asterisk (*) next to the datapoint.

The anion and osmolal gaps from the initial laboratory studies of patients with detectable ethylene glycol, isopropanol, and methanol by GC are shown in Figure 3. Laboratory data necessary to calculate anion gap were not available for 4 patients (3 with OG > 14) with detectable toxic alcohols; thus, these 4 patients are not included in Figure 3 (leaving a total of 80 patients). All patients that tested positive for ethylene glycol, isopropanol, propylene glycol, or methanol had an elevated OG or anion gap or both, keeping in mind there was one patient with detectable ethylene glycol by GC who initially presented with an OG of 6 but did not have laboratory data sufficient to calculate anion gap (Table 2).

In the 84 patients who had toxic alcohols detected by GC, 54 (64.3%) had a single toxic alcohol detected without co-ingestion of ethanol or another toxic alcohol. Twenty-nine patients (34.5%) had both ethanol and one toxic alcohol detected. In patients ingesting both ethanol and either methanol or ethylene glycol, the presence of ethanol could inhibit the metabolism of the toxic alcohol by alcohol dehydrogenase and thereby limit formation of toxic metabolites. Only one patient had two toxic alcohols detected; this was a 52 year old male who ingested "cleaning solutions" in a suicide attempt and for whom GC analysis detected both isopropanol (18 mg/dL) and methanol (10 mg/dL).

There were 7 patients for whom propylene glycol was the suspected primary cause of the elevated OG, with serum propylene glycol concentrations ranging from 45 to 147 mg/dL and no other toxic alcohol detected. In 6 of these 7 patients, the increased OG was entirely explained by the presence of propylene glycol, using the propylene glycol serum concentration (in mg/dL) divided by 7.2 as the estimated osmolal contribution [39]. In one patient, there was still an OG of 15 following correction for the estimated osmolar contribution of the propylene glycol. Only three of the seven patients with propylene glycol had an anion gap of 16 or greater on initial laboratory studies (Figure 3). Two of the seven patients had been administered multiple doses of lorazepam intravenously, a formulation that contains propylene glycol (up to 80% v/v) as the diluent, prior to laboratory studies. Five patients had been administered activated charcoal preparations containing propylene glycol at an outside hospital prior to transfer; none of these five patients had a documented history of being administered any medications containing propylene glycol as the diluent prior to the blood draw used for OG and propylene glycol determinations.

### Causes of elevated OG other than toxic alcohols ("unexplained osmolal gaps")

Through detailed chart review we attempted to assign the most likely primary cause for the 264 patients who had elevated OG (> 14) without detection of toxic alcohols on initial laboratory studies (Figure 2). The most common suspected causes of elevated OG in the 264 patients were ethanol-related (including alcoholic ketoacidosis) (n = 72, 27.2%), renal failure (n = 43, 16.3%), diabetic ketoacidosis (n = 33, 12.5%), shock (n = 32, 12.1%), and therapeutic infusion of mannitol (n = 15, 5.7%). In 69 patients (26.1%), none of the well-established causes of elevated OG were found (classified in category of "Other" in Figure 2).

For the 72 patients classified in the "ethanol-related" category, all had recent history of binge drinking (with risk of alcoholic ketoacidosis) and an absence of clinical suspicion for toxic alcohol ingestions. Fifty-three of these 72 patients had detectable serum ethanol but still showed elevated OG (> 14) after correction for the estimated osmolal contribution of ethanol. Alcoholic ketoacidosis is characterized by high serum ketone levels and elevated anion gap [1,48,49], typically in the setting of recent binge drinking with limited nutritional intake. However, diagnosis of alcoholic ketoacidosis is sometimes only inferred because laboratory analyses such as serum ketones may not be obtained. Even for patients who clearly have the characteristics of alcohol ketoacidosis, the diagnosis assigned to the patient in the medical record may often not be alcoholic ketoacidosis but a more general diagnosis such as ethanol withdrawal or intoxication.

For the 72 patients in our study with elevated OG suspected to be related to ethanol, a breakdown of the laboratory studies performed in the "ethanol-related" group is included in Additional File 1. Forty-four of the 72 patients had serum ketones above upper limit of reference range and/or positive urine ketones. An additional three patients had elevated anion gap (16 or greater) but no laboratory analysis of ketones. Thus, 47 patients had some laboratory characteristic of alcoholic ketoacidosis.

For the patients that had elevated OG due to a cause other than ingestion of toxic alcohols, 12.2% had an OG greater than 30. Only 3.2% of these patients had OG greater than 50, and none had OG greater than 100. In contrast, for patients ingesting toxic alcohols, 48.7% had OG greater than 30 on initial laboratory work-up and 19.7% had OG greater than 50. All cases where OG was greater than 80 in the absence of toxic alcohols were patients with a history of heavy recent ethanol use and possible alcoholic ketoacidosis. The highest OG values in patients without history of ethanol or toxic alcohol use were 60 (42 year old male given mannitol for increased intracranial pressure approximately one hour prior to blood draw) and 51 (39 year old female with fulminant liver failure due to massive acetaminophen overdose two days prior to admission).

For the 72 cases where recent heavy ethanol use was the suspected main cause of the elevated OG due to alcoholic ketoacidosis, 19 of the patients had no detectable plasma ethanol at time of presentation to the hospital but did have history of recent heavy consumption of ethanol and were admitted to the hospital for management of ethanol withdrawal symptoms. Overall, 63 of the 72 patients whose elevated OG was suspected to be due to recent heavy ethanol use (in the absence of toxic alcohols) had OG values between 14 and 30. For the 7 patients with OGs greater than 30 but no toxic alcohols detected, five had detectable serum ethanol (ranging from 115 to 313 mg/dL). Of the 15 cases where mannitol was the suspected cause of elevated OG, 14 had OG of 33 or less.

### Toxic alcohol ingestions presenting without elevated OG

We found 7 instances where a patient had OG of 14 or less on initial laboratory analysis had GC analysis detect the presence of toxic alcohols (summarized in Table 2). Six of 7 of these patients were positive for ethylene glycol, with clinical histories documenting varying amounts of ethylene glycol ingestions ("4 oz", "8 oz", "1 cup", "4-5 mouthfuls", "accidental" ingestion while trying to siphon antifreeze by mouth from radiator, and an unknown quantity in one case). One patient was a 28 month old toddler that drank an unknown quantity of windshield fluid containing methanol and was brought to emergency department within 10 minutes of ingestion. This child had an anion gap of 16. Six of 7 patients had anion gaps of 15 or greater. Laboratory values needed to determine anion gap were not ordered for one patient.

### Diagnostic performance of OG

**Figure 4:**
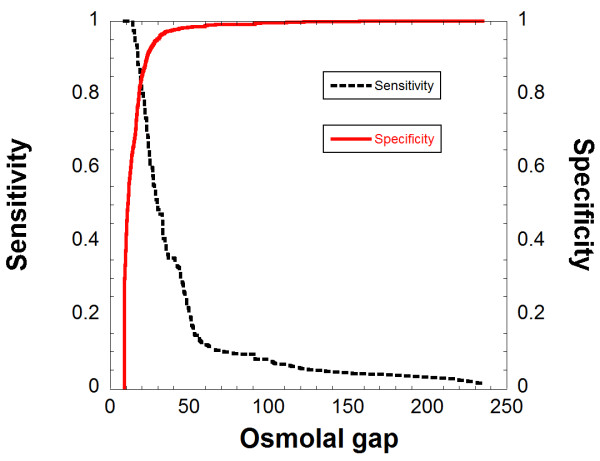
**Sensitivity and specificity for osmolal gaps for diagnosis of toxic alcohol ingestions**. The analysis includes all patients with OG of 9 or greater.

**Figure 5:**
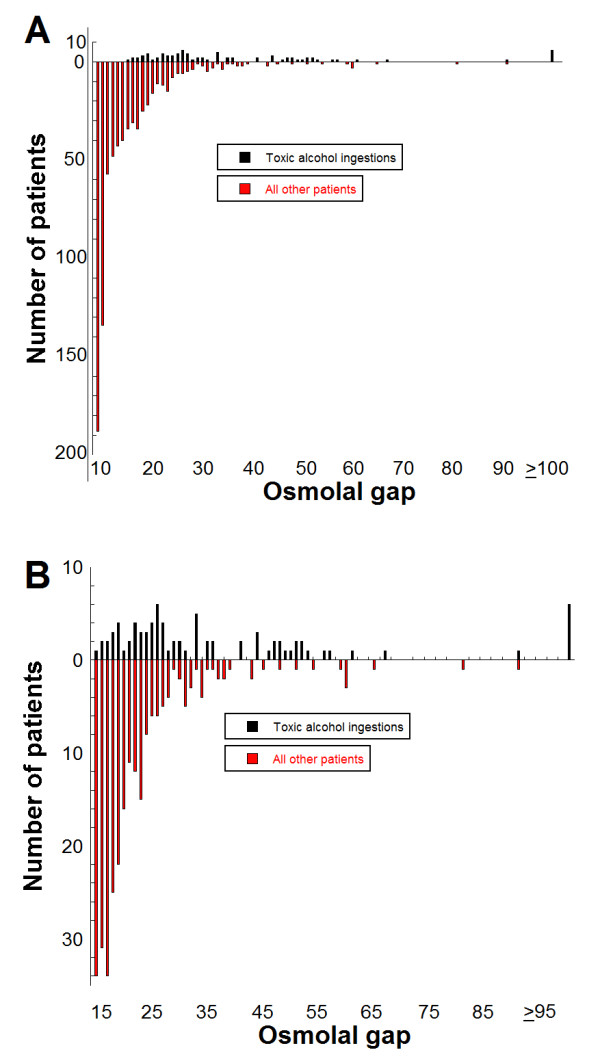
**Gerhardt plots for osmolal gaps for diagnosis of toxic alcohol ingestions**. The bars above the horizontal line at 0 indicate number of patients identified as having ingested toxic alcohols at a particular OG. The bars below the horizontal line indicate number of patients with a cause other than toxic alcohol ingestion at that particular OG. The plot in (A) shows all data for OG of 9 and above. The plot in (B) focuses on data for OG 15 and above only.

We additionally performed chart review for 510 patients who had OG between 9 and 14 on their initial laboratory studies. Within this group, 39 patients had GC analysis; all were negative for presence of toxic alcohols by GC or for any clinical history compatible with toxic alcohol ingestion. Using data from all patients who had OG of 9 or greater on initial laboratory studies (total n = 851), Figure 4 shows plots of sensitivity and specificity for different OG cutoffs. At a cutoff of 20, OG has a sensitivity of 0.82 (95% confidence interval, CI, 0.71-0.89) and specificity of 0.85 (95% CI, 0.82-0.87). At an OG cutoff of 30, the sensitivity is only 0.49 (95% CI, 0.38-0.60) with a specificity of 0.95 (0.94-0.97). Figure 5 shows a Gerhardt plotting the number of patients who either were shown to have ingested toxic alcohols by GC (above y = 0 horizontal line) or did not show toxic alcohol ingestions by GC and/or clinical chart review (below y = 0 horizontal line).

## Discussion

This study demonstrates that the OG has a sensitivity of 0.82 and a specificity of 0.85 at a cutoff of 20 for diagnosing toxic alcohol ingestion. These findings are similar to those of another recent study [22]. The most common suspected reasons for elevated OG in the absence of toxic alcohol ingestion (in descending order of frequency) were suspected alcoholic ketoacidosis, renal failure, shock, diabetic ketoacidosis, and recent administration of mannitol. When the elevated OG was due to something other than toxic alcohol ingestion, the gap was typically less than 30. For the 264 patients that had elevated OG due to a cause other than ingestion of toxic alcohols, only 12.2% had an OG greater than 30. Exceptions included patients with alcoholic ketoacidosis and recent mannitol infusion, which can present with markedly elevated OG depending on timing of laboratory analysis. In our study, all patients that had toxic alcohols detected by GC had an osmolal gap and/or anion gap.

Estimation of the contribution of ethanol to OG is a complicated and controversial subject with multiple formulae proposed [18,26-28,50] One challenge is that the compounds formed in alcoholic ketoacidosis (e.g., glycerol, acetone, acetal, and 1,2-propanediol) may contribute to osmolality but are not directly measured by routine clinical laboratory testing [23,30-32]. In our study, 19 patients had elevated OG that by clinical history was most likely due to recent heavy ethanol ingestion but with serum ethanol less than 10 mg/dL.

Our study was able to detect cases of elevated OG possibly due to administration of activated charcoal. In the United States, there are multiple formulations of activated charcoal, some of which contain propylene glycol as the excipient. We found 5 cases where GC analysis demonstrated detectable propylene glycol (in one case as high as 147 mg/dL) following activated charcoal administration. Although there have been many studies of the antidotal properties of activated charcoal, no pharmacokinetic study in humans has examined the OG that may be caused by the propylene glycol excipient. A pharmacokinetic study of activated charcoal has been performed on dogs showing substantial increases in serum osmolality and lactate following administration of activated charcoal doses typical of veterinary practice [51]. A study of the pharmacokinetics of activated charcoal in humans with respect to OG and propylene glycol absorption would be of interest.

This study has several limitations. The first is that clinical and laboratory practice for the medical center of this study utilized OG (specifically greater than 14) as a major factor in determining whether GC analysis was performed, resulting in a potential work-up bias. In particular, although GC analysis could be performed in the absence of an elevated OG with pathology resident or attending physician approval, the extra approval step could have acted as barrier to getting GC analysis performed. This raises the possibility that toxic alcohol ingestions were missed by clinical history and physical but may have been detectable by GC analysis had it been performed. However, chart review was performed on all patients with OG of 9 to 14 (comprising 510 patients that was in addition to the 341 patients with OG greater than 14), which did not reveal additional patients with clinical histories compatible with toxic alcohol ingestion. This suggests that clinically significant ingestions of toxic alcohols rarely present without an elevated OG.

The second limitation is that interpretation of OG is often made in the absence of a prior 'baseline' OG measured during a time when the patient is not ill or not intoxicated. For example, an OG of +10 (within the reference range of many clinical laboratories) could be clinically meaningful in a patient whose baseline OG (during time of health) is -10. Further complicating interpretation of OG is that reference ranges for OG vary across clinical laboratories based on institutional practice and the formula used to calculate OG. This can be an issue particularly when patients are transferred from one hospital to another.

The third limitation is that the study population includes many patients who were transferred from other hospitals in addition to patients who presented for initial diagnosis. For example, for patients who have ingested ethylene glycol or methanol, an OG is seen early in ingestion while an anion gap (and a declining OG) appears as the parent compound is metabolized. Transfer patients can also present a referral bias in that patients with toxic alcohol ingestions may be preferentially transferred to tertiary care centers as opposed to other patients presenting with other causes of altered mental status.

## Conclusions

Our study concurs with other investigations that show that OG can be a useful diagnostic test in conjunction with clinical history and physical examination. Clinicians should be aware of the common causes of elevated OG other than toxic alcohols such as alcoholic ketoacidosis, renal failure, shock, and diabetic ketoacidosis, and recent administration of mannitol. In addition, given the technical challenges of GC analysis, sensitive and specific rapid screening tests (e.g., enzymatic assays) for ethylene glycol and methanol would be useful.

## Competing interests

The authors declare that they have no competing interests.

## Authors' contributions

MDK was involved in the study concept and design, analysis and interpretation of the date, drafting and revisions of the manuscript. RMW and JM both contributed to the study design, data analysis, and critical interpretation. All authors have read and approval the final manuscript.

## Pre-publication history

The pre-publication history for this paper can be accessed here:

http://www.biomedcentral.com/1472-6890/12/1/prepub

## Supplementary Material

Additional file 1 **Summary of laboratory findings in patients with ethanol-related cause of elevated osmolal gap**. Contains a breakdown of the laboratory studies performed in patients with elevated osmolal gap thought to be related to recent ethanol consumption. Click here for file
