# Supplementary material for: A retrospective analysis of glycol and toxic alcohol ingestion: utility of anion and osmolal gaps
Source: BMC Clin Pathol. 2012 Jan 12;12:1. doi: 10.1186/1472-6890-12-1 (PMC3281782; doi:10.1186/1472-6890-12-1)
Supplement: Additional file 1 — Summary of laboratory findings in patients with ethanol-related cause of elevated osmolal gap. Contains a breakdown of the laboratory studies performed in patients with elevated osmolal gap thought to be related to recent ethanol consumption. [file 1472-6890-12-1-S1.DOC]

Additional file 1

Summary of laboratory findings in patients with ethanol-related cause of elevated osmolal gap

| **Laboratory study** | **Number of patients with elevated value above upper limit of reference range** | **Total number of times ordered** |
| --- | --- | --- |
| Serum ketones | 30 | 46 |
| Urine ketones | 26 | 44 |
| Anion gap | 31 | 66 |

Serum ketones + urine ketones both ordered (n=36)

Only serum ketones elevated (n=12)

Only urine ketones elevated (n=8)

Both serum and urine ketones elevated (n=12)

Neither serum nor urine ketones elevated (n=4)

Only serum ketones ordered (n=10)

Serum ketones elevated (n=6)

Serum ketones within reference range (n=4)

Only urine ketones ordered (n=8)

Urine ketones elevated (n=6)

Urine ketones negative (n=2)

Total patients with elevated serum and/or urine ketones (n=44)

Number with concomitant anion gap of 16 or greater (n=28)

Neither serum nor urine ketones ordered (n=18)

Number with anion gap of 16 or greater (n=3)
